# The value of manure - Manure as co-product in life cycle assessment

**DOI:** 10.1016/j.jenvman.2019.03.059

**Published:** 2019-07-01

**Authors:** Adrian Leip, Stewart Ledgard, Aimable Uwizeye, Julio C.P. Palhares, M. Fernanda Aller, Barbara Amon, Michael Binder, Claudia M.d.S. Cordovil, Camillo De Camillis, Hongming Dong, Alessandra Fusi, Janne Helin, Stefan Hörtenhuber, Alexander N. Hristov, Richard Koelsch, Chunjiang Liu, Cargele Masso, Nsalambi V. Nkongolo, Amlan K. Patra, Matthew R. Redding, Mariana C. Rufino, Ruben Sakrabani, Greg Thoma, Françoise Vertès, Ying Wang

**Affiliations:** a European Commission, Joint Research Centre, Ispra, VA, Italy; b Farm Systems & Environment Group, AgResearch, Private Bag 3123, Hamilton, New Zealand; c Food and Agriculture Organization of the United Nations, Animal Production and Health Division, Rome, Italy; d Animal Production Systems Group, Wageningen University & Research, PO Box 338, 6700 AH,, Wageningen, the Netherlands; e Teagasc – Crops, Environment and Land Use Programme, Johnstown Castle, Wexford, Y35 Y521, Ireland; f Environmental Impacts and Water Management in Livestock, Embrapa Southeast Livestock, São Carlos, SP, Brazil; g I.S.Environment, UK; h Leibniz Institute for Agricultural Engineering and Bioeconomy (ATB), Germany and University of Zielona Góra, Faculty of Civil Engineering, Architecture and Environmental Engineering, Poland; i Evonik Nutrition&Care GmbH, Germany; j University of Lisbon, Instituto Superior de Agronomia, LEAF, Lisboa, Portugal; k Institute of Environment and Sustainable Development in Agriculture, Chinese Academy of Agricultural Sciences, Beijing, 100081, China; l Key Laboratory of Energy Conservation and Waste Treatment of Agricultural Structures, Ministry of Agriculture, Beijing, 100081, China; m The University of Manchester, School of Chemical Engineering and Analytical Science, UK; n Natural Resources Institute Finland, Unit of Bioeconomy and Environment, Helsinki, Finland; o Research Institute of Organic Agriculture FiBL, Vienna, Austria; p The Pennsylvania State University, USA; q University of Nebraska - Lincoln, USA; r School of Agriculture and Biology, Shanghai Jiao Tong University, PR China; s International Institute of Tropical Agriculture, Nkolbisson, Messa, Yaounde, Cameroon; t ARC-Institute for Soil, Climate and Water, South Africa; u Dept of Agriculture and Animal Health, UNISA, South Africa; v IFA-Yangambi, Dem. Rep. Congo; w West Bengal University of Animal and Fishery Sciences, Department of Animal Nutrition, Kolkata, India; x Queensland Department of Agriculture and Fisheries, Australia; y Lancaster University, Lancaster Environment Centre, Lancaster, UK; z School of Water, Energy & Environment, Cranfield University, United Kingdom; aa Ralph E. Martin Department of Chemical Engineering, University of Arkansas, USA; ab UMR SAS, INRA, Agrocampus Ouest, 35000 Rennes, France; ac Innovation Center for U.S. Dairy, USA

**Keywords:** Life cycle assessment, Livestock supply chains, Nutrients, Fertilizer, Allocation, Manure

## Abstract

Livestock production is important for food security, nutrition, and landscape maintenance, but it is associated with several environmental impacts. To assess the risk and benefits arising from livestock production, transparent and robust indicators are required, such as those offered by life cycle assessment. A central question in such approaches is how environmental burden is allocated to livestock products and to manure that is re-used for agricultural production. To incentivize sustainable use of manure, it should be considered as a co-product as long as it is not disposed of, or wasted, or applied in excess of crop nutrient needs, in which case it should be treated as a waste. This paper proposes a theoretical approach to define nutrient requirements based on nutrient response curves to economic and physical optima and a pragmatic approach based on crop nutrient yield adjusted for nutrient losses to atmosphere and water. Allocation of environmental burden to manure and other livestock products is then based on the nutrient value from manure for crop production using the price of fertilizer nutrients. We illustrate and discuss the proposed method with two case studies.

## Highlights

• New method to value manure for its beneficial effects on crop production is proposed.
• Manure can be considered as a co-product in Life Cycle Assessments when applied according to crop nutrient requirements.
• The method encourages farmers to use manure sustainably in livestock and crop production systems.

## 1 Introduction

The livestock sector contributes to the livelihood of millions of people, but their production poses several environmental challenges such as greenhouse gas emissions, eutrophication, acidification and biodiversity loss (Mottet et al., 2017). While livestock products are rich in essential macro- (e.g. proteins) and micronutrients (e.g. Zn, Fe, Vitamins, see Parodi et al., 2018) contributing globally 18% of food energy and 25% of food protein (Steinfeld et al., 2006) the consumption of livestock products can also be associated with health risks (Springmann et al., 2018; Willett et al., 2019). Livestock can help transfer and convert proteins from plant biomass (e.g. grass or by-products, rangelands, or food waste) into animal-sourced foods utilizing resources that otherwise cannot be consumed by humans, but in other cases livestock production is in competition with other land uses such as food, fiber and energy production (Van Zanten et al., 2018).

Ruminants, pigs and poultry have very different nutritional requirement for crude protein and energy, feed conversion efficiencies, and pathways of N excretion in manure. Feed N conversion efficiencies are highest for pigs and poultry, and lowest with beef cattle and depend on the type and amount of N consumed by each particular livestock species (Flachowsky, 2002; Huhtanen and Hristov, 2009). In general terms, between 55 and 95% of the nitrogen (N) and about 70% of the phosphorus (P) ingested by livestock are excreted through urine or feces (Menzi et al., 2010). A further inefficiency can occur from imbalances between nutrient imports (e.g. purchased feeds, animals) and managed exports (e.g. sale of animal products, manure) in livestock production systems, resulting in nutrient losses to the environment and additions to soil storage (Waldrip et al., 2015).

The management of nutrient supply for agricultural production is central to agriculture and food supply chains. It has driven the development of agricultural practices through time, such as manure recycling and crop residues management, as well as the application of mineral fertilizer since its invention in the early 20th century (Erisman et al., 2008; Gerber et al., 2014). The considerable perturbation of the nutrient cycles since the industrialization of agriculture through increased fertilizer use and agricultural production has amplified the detrimental effects on ecosystems and human health (Galloway et al., 2003; Leip et al., 2015; Sutton et al., 2011). There is great uncertainty on how best to strike a balance between fertilizer (and manure) nutrient recommendations, economic yield, nutrient use efficiency (NUE) and environmental outcomes (Dalgaard et al., 2014). The nutrient value of manure has long been recognised but the ease of use of inexpensive manufactured mineral fertilizers has led to their dominance in many industrialized countries (Powell et al., 2010).

Life cycle assessment (LCA) is a widely used tool to assess the environmental impacts of livestock supply chains and of resultant products (e.g. Gerber et al., 2010; van Zanten et al., 2016; Weiss and Leip, 2012). Where multiple products are produced, the environmental emissions are allocated between the various co-products, usually recognizing the stepwise procedure outlined in ISO14044 (ISO, 2006). When manure is produced as an output from livestock systems it may be considered as a waste or residual, where all system emissions are assigned to the other products, or as a co-product where it is recognised as a valuable product. For example, in the Livestock Environmental Assessment and Performance (LEAP) partnership guidelines on poultry (FAO, 2016a) and large ruminants (Fao, 2016b) it is recommended that if manure is a valuable co-product, the production system emissions are allocated using a biophysical approach based on the energy for digestion that is generated by the animal for production of the manure. Economic allocation based on the relative revenue received for manure compared with that for the other co-products at the farm-gate could be a viable alternative. In practice, however, the economic revenue from manure may be an artifact of regulatory policy and may not be a good representation of the true value of the manure, depending on the geographical context. Manure also has value to improvements in soil quality and productivity, for example in building up organic matter or improving soil water retention, which often are difficult to quantify and are variable based upon past soil management practice. In most cases, manure is used as a valuable resource because of its nutrient value, particularly a source of the major nutrients of N and P, none of the current approaches is able to properly reflect this benefit that drives farmers to accept manure. Thus, an alternative approach for allocating emissions from a manure co-product could be based on its nutrient value.

The objective of this study is to present a new methodology for the allocation of emissions in livestock supply chains over the co-products, based on the nutrient value of manure for crop production. In Section 2 we first introduce the concept and a method called ‘theoretical approach’. As data for this approach will in most cases be unavailable we simplify the approach in Section 2.2 (‘pragmatic approach’). The concept is illustrated using two case studies introduced in Section 2.3-2.4 with the results presented in Section 3 and discussed in Section 4. We finally conclude in Section 5.

This study was developed in the context of the technical advisory group on modelling of nutrient flows and impact assessment in the livestock supply chains (FAO, 2018a, FAO, 2018b) of the Livestock Environmental Assessment and Performance (LEAP) Partnership. The LEAP Partnership is a multi-stakeholder initiative composed of three stakeholder clusters: Governments, Private Sector, and Civil Society and Non-Governmental Organizations and is hosted by the Food and Agriculture Organization of United Nations (FAO).

## 2 Methods

In the following, we develop a method for the quantification of the allocation of upstream emissions from a livestock system *A* between manure and other livestock products using attributional LCA, where the manure is used outside this livestock system. We do so based on the value that the manure provides to a system *B* which can be another livestock system, a crop system, or a non-agricultural system. As such, the method could be described as a ‘hybrid’ allocation approach, since it looks beyond the point of allocation as done in system expansion approaches. We note that this manuscript will look at the value that is carried with nutrients in manure thus ignoring other possible benefits of using manure. However, the approach described in this study can easily be extended to capture further benefits.

### 2.1 Theoretical approach

#### 2.1.1 Fertilizer equivalent

To estimate the fertilizer value of manure, a framework based on plant growth curves is proposed as a basis to allocate environmental emissions from animal supply chains between the main animal products and manure. Most farmers use manure as an organic fertilizer because of its availability but the specific nutrient equivalent of the manure is often not estimated. This fertilizer equivalence defines the amount of manure that the farmer would apply if s/he had to purchase mineral fertilizers to provide the required nutrients (e.g. N and P) for plant uptake. Because manure is generally not traded with a price based on its fertilizer properties, the fertilizer equivalence value needs to be estimated with other approaches. For nutrients, this can be measured using the synthetic mineral fertilizer that the farmer would buy in case the manure was not available.

For mineral fertilizers, the ‘economic optimum’ describes the application rate at which the marginal cost of additional fertilizer applications is the same as the additional revenue from increased harvest. If the cost for the mineral fertilizer is $C_{min,nut}$ [US$ (kg nutrient)^−1^] and revenue for the harvest is $R_{crop}$ [US$ (kg DM)^−1^], then the economic optimum *ecopt* is the application rate of mineral fertilizer $Q_{min,nut}$ [kg nutrient ha^−1^yr^−1^] at which Equation (1) holds:

(1) $${\partial Y_{crop}|}_{ecopt}\cdot R_{crop}={\partial Q_{min,nut}|}_{ecopt}\cdot C_{min,nut}$$

where $Y_{crop}$ [kg DM ha^−1^yr^−1^] is the yield of a crop. For a full list of symbols and indices used throughout the paper please see Section 7.

For higher fertilizer application rates, the costs become higher and it is thus not rational to apply beyond the economic optimum from an agronomic perspective. However, if nutrients are free of costs, additional application may lead to yield increases until the physical optimum, beyond which application of fertilizers will not add any benefit.

The nutrient equivalent $f_{eq}$ for the nutrients contained in the manure defines the amount of mineral fertilizers that provides the same amount of nutrients to the crop. Here we are interested in the amount of manure that provides the same crop uptake as mineral fertilizers at the economic optimum for mineral fertilizers *ecopt*. We define therefore the nutrient equivalent $f_{eq}$ as the ratio of mineral fertilizer application at the economic optimum and manure application rate providing the same uptake of nutrients (Equation (2)).

We define useful outputs (*Q*_*output,nut*_*)* as the nutrients taken up by the plant biomass, including crop residues (*Q*_*plant,nut*_*)*, plus possible accumulation of the nutrient in the soil if ultimately available for crop uptake (*Q*_*ssc,nut*_). The difference between all inputs and useful outputs of the nutrient *nut* ($Q_{input,nut}-Q_{output,nut}$) gives the nutrient surplus ($Q_{surplus,nut}$) which equals the sum of all losses to atmosphere and hydrosphere (see the nutrient balance Equation (3)). Assuming equal distribution of nutrients across crop compartments (harvested crops, straw, crop residues, roots), the only difference in N output between different fertilizer types is the soil stock change (*Q*_*ssc*_).

(2) $$f_{eq,nut}=\frac{Q_{min,nut,ecopt}}{Q_{man,nut,ecopt}}$$

(3) $$Q_{input,nut}=Q_{plant,nut}+Q_{ssc,nut}+Q_{surplus,nut}$$

**Fig. 1:**
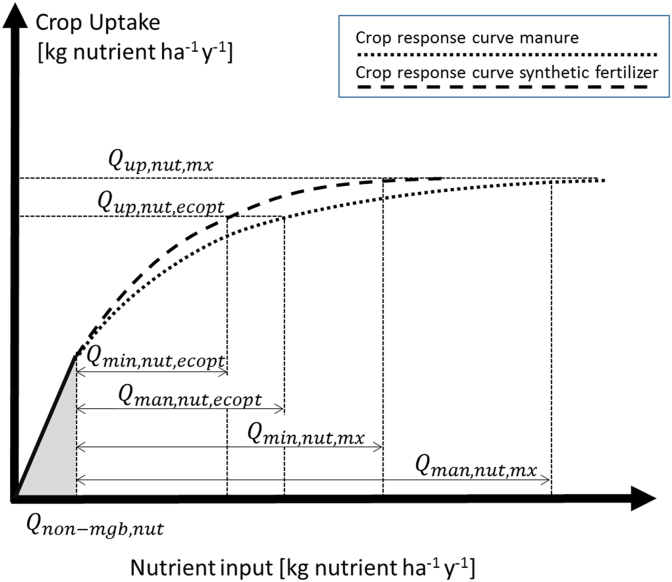
Illustration of crop response curves for increasing application rates of nutrients from mineral fertilizers and manure. This assumes a lower NUE for manure than for mineral fertilizers. In both cases, input of fertilizer is given in addition to non-manageable nutrient inputs from atmospheric deposition and biological N fixation, or mineralization of e.g. crop residues. The figure shows the location of the economic optimum (at rates for ecopt) and the physical optimum at rates mx.

The concept is illustrated in Fig. 1 where a higher nutrient use efficiency (NUE) is assumed for mineral fertilizer as compared to manure.

### 2.2 Value of manure

The value of the applied manure as a co-product is calculated from the amount of manure nutrients provided with manure up to yield that is achieved at the economic optimum for mineral fertilizers, net of nutrients that are obtained from other sources such as net soil mineralization, biological nitrogen fixation, or atmospheric deposition. This is to emphasize sustainable use of those nutrient sources. We summarize them in the term ‘non-manageable nutrient input’ (*Q*_*non-mgb*_) (Equation (4)). Note that because of the convention of considering soil mineralization (=depletion of soil nutrient stocks) as a negative output, $Q_{ssc,nut}$ is used in Equation (4) with a negative sign.

(4) $$Q_{non-mgb,nut}=Q_{bnf,nut}+Q_{atmdep,nut}-Q_{ssc,nut}$$

with *ssc:* soil stock changes*, bnf:* biological fixation of atmospheric nitrogen*, atmdep:* atmospheric deposition*.* For P, this would include P release from bedrock.

Nutrients from land-applied manure and compost continue to become plant-available in successive growing seasons (Bar-Tal et al., 2004; Hadas et al., 1996; Hanč et al., 2008). Equation (6) allows mineralization of residual manure-nutrient to be accounted for by increasing the input of *Q*_*ssc,nut*_ to *Q*_*non-mgb,nut*_. This shifts the crop response curves for manure and mineral fertilizers (Fig. 1) to the right, decreasing the requirement for nutrient inputs to meet current season demand. A range of models are available that enable this manure nutrient mineralization and availability to be estimated (e.g. Archontoulis et al., 2014; Beraud et al., 2005).

In case the farmer applies manure at a rate beyond $Q_{man,nut,ecopt}$ (see Equation (2)) when the response rate is declining but below the physical maximum $Q_{man,nut,MX}$ s/he generates value only because the manure is freely available (or cheaper than mineral fertilizers) and external costs caused by the losses are not internalized. This share of manure must be considered as a co-product but using a lower value.

The amount of manure valued as a co-product with the full fertilizer cost $C_{min,nut}$ is calculated as $Q_{full,nut}$ in Equation (5). It is the manure application rate at the economic optimum, or the total application rate of nutrients in manure if this is less than the application rate at the economic optimum.

Equation (6) calculates the additional manure$Q_{low,nut}$, that is valued as a co-product but with lower nutrient equivalent price. $Q_{low,nut}$ is the difference of the manure application rate and $Q_{full,nut}$ , if positive – but not more than the difference between $Q_{man,mx}$ and $Q_{full,nut}$. The lower nutrient price is calculated from the integral of the additional benefit of manure application, being the nutrient equivalent value close to the economic optimum, and zero at the physical maximum, because no further yield increase results from the application. The lower nutrient price is therefore approximately half fertilizer price if $Q_{low,nut}$ is at the level or higher than at the physical maximum $Q_{man,mx,nut}$. Generalizing, we use a discount factor for calculating the total value of the applied manure $V_{man,nut}$ in Equation (7).

(5) $$Q_{full,nut}=\min\left\{Q_{man,nut,ecopt},Q_{man,nut}\right\}$$

(6) $$Q_{low,nut}=\max\left(0,\min\left(Q_{man,nut,mx},Q_{man,nut}\right)-Q_{full,nut}\right)$$

(7) $$\begin{array}{cc} V_{man,nut} & \;=Q_{coprd,nut}\cdot f_{eq}\cdot C_{min,nut}\\ & \;\;\;\;\;\;\;=\left(Q_{full,nut}+Q_{low,nut}\cdot f_{discount}\right)\cdot f_{eq}\cdot C_{min,nut} \end{array}$$

In the case where multiple nutrients are assessed, the benefit of each nutrient is evaluated separately, and the values assumed to be additive in order to estimate the total value of the applied manure. Hence, in an example where both N and P are being evaluated, the total value of manure would be according to Equation (8):

(8) $$V_{man}=V_{man,N}+V_{man,P}$$

Any application of nutrients in manure beyond $Q_{man,mx,nut}$ is considered as waste ($Q_{waste}$) and all associated emissions are allocated back to the livestock supply chain that produced the manure. The method for assigning a value ($Q_{man,nut})$ vs. waste ($Q_{waste}$) for nutrients applied in excess should be nutrient specific.

(9) $$Q_{waste,nut}=\min\left(0,Q_{man,nut}-Q_{man,mx,nut}\right)$$

**Fig. 2:**
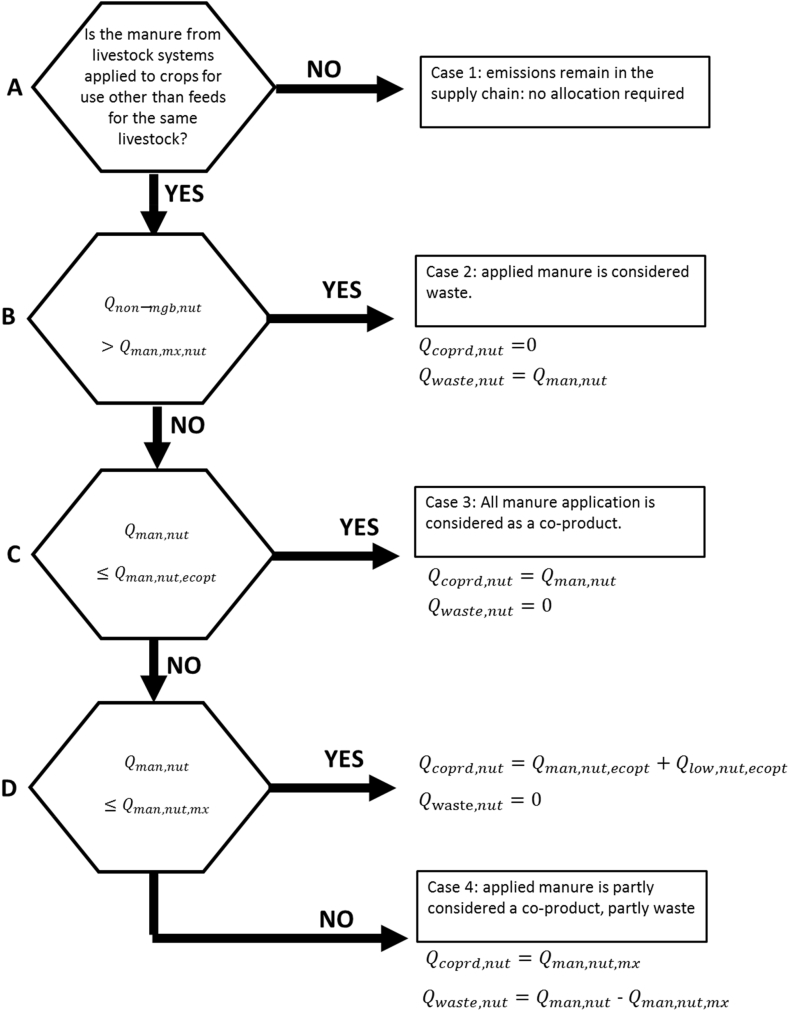
A decision diagram illustrating the possible share of applied manure that is considered a co-product or a waste. For explanation of the symbols see text.

Fig. 2 illustrates the possible cases for determining the amount of applied manure that is considered as a co-product or waste, or ‘remains’ within the same livestock supply chains.

#### 2.2.1 Allocation of emissions

To allocate the emissions from the livestock supply chain $E_{lvst}$ [*kg CO_2-eq_*] for manure versus other co-products (e.g. live-animals, eggs, wool, milk), the economic value is used. These emissions arise from the animals (e.g. enteric fermentation), housing, and manure treatment and management up to the point that it or a part of it is sold to a crop farmer, and all emissions from manure that is used within the supply chain (the ‘NO’ branch at level A in Fig. 2). The livestock co-products return a revenue to the farmer of $V_{lvst}$ [US$]. But a part of the manure is exported out of the supply chain for application to croplands or other uses. That manure can cause further emissions $E_{man}$ [*kg CO_2-eq_*] at the point where it is stored and following application to the fields.

Generalizing Equation (8) for a situation where the manure is applied on different fields *i* at a share $x_{i}$, the total value of applied manure is obtained as in Equation (10):

(10) $$V_{man}=\underset{i}{\sum }\underset{nut}{\sum }x_{i}\cdot V_{man,nut,i}$$

The allocation of emissions to the livestock products ($\alpha_{lvstk}$) and manure ($\alpha_{man})$ supply chains is according to economic allocation thus:

(11) $$\begin{array}{l} \alpha_{lvstk}=\frac{V_{lvstk}}{V_{man}+V_{lvstk}}\\ \alpha_{man}=\frac{V_{man}}{V_{man}+V_{lvstk}} \end{array}$$

The total emissions to be allocated amongst the livestock products include also any emissions on the crop farm for any of the applied manure that is considered as a waste:

(12) $$E_{lvstk\_total}=E_{lvstk}+E_{man}\cdot \frac{\sum_{i}x_{i}\cdot Q_{waste,i}}{Q_{man}}$$

For whole farm system analysis, allocation between the various livestock products (e.g. milk, meat, fibre) would be carried out using recommended protocols (e.g. FAO, 2016a, FAO, 2016b IDF, 2015).

### 2.3 Pragmatic approach

In many situations, available information is insufficient to establish a crop response curve and NUE at the economic optimum for mineral fertilizers and/or manure are not available. In those cases, we propose to estimate the nutrient equivalent $f_{eq}$ on the basis of *actual* crop nutrient uptake rates and *standard* loss rates, using e.g. default loss rates as in the IPCC (IPCC, 2006) or LEAP (FAO, 2018a, FAO, 2018b) guidelines, taking into consideration environmental conditions and farm management practices as far as possible, or using representative loss rates measured or modelled for representative/similar conditions. These conditions can be used to estimate standard mineral nutrient $\left(Q_{min,nut,standard}\right)$ and manure nutrient $\left(Q_{man,nut,standard}\right)$ application rates. Thus, Equation (2) transforms to:

(13) $$f_{eq,nut}=\frac{Q_{min,nut,standard}}{Q_{man,nut,standard}}$$

Assuming losses to the atmosphere and to the hydrosphere, both expressed as fractions of available nutrient that is lost through the respective pathways, the nutrient equivalent is obtained from Equation (14).

(14) $$f_{eq,nut}=\frac{1-\left(Frac_{atm,man,nut}+Frac_{hyd,man,nut}-Frac_{atm,man,nut}\cdot Frac_{hyd,man,nut}\right)}{1-\left(Frac_{atm,min,nut}+Frac_{hyd,min,nut}-Frac_{atm,min,nut}\cdot Frac_{hyd,min,nut}\right)}$$

whereby the term $Frac_{atm,min,nut}\cdot Frac_{hyd,min,nut}$ (and the corresponding term for manure) is required to account for the fact that nutrients available for potential loss to the hydrosphere represent the net after subtracting the losses to the atmosphere. This is the case when losses occur mainly through leaching rather than run-off processes.

**Fig. 3:**
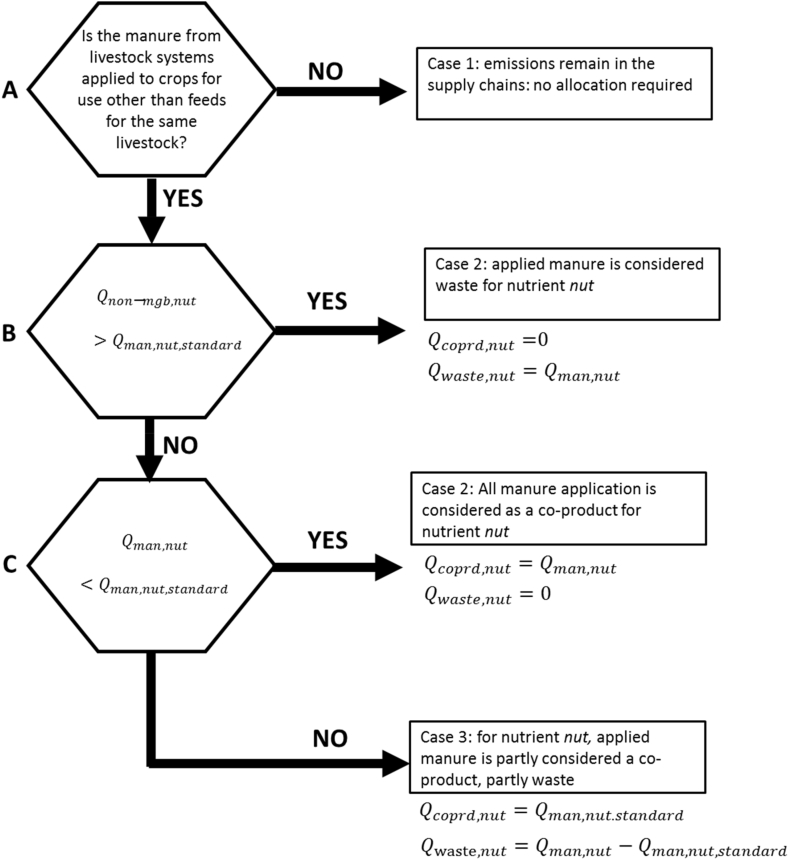
A decision diagram illustrating the possible share of applied manure that is considered a co-product or a waste following the pragmatic approach. For explanation of the symbols see text.

In the pragmatic approach, the standard application rate is estimated from the difference between crop uptake rate and losses. It is not possible to distinguish between the economic and physical optimum and the decision diagram simplifies to that in Fig. 3. Thus, it is different from the theoretical approach in not directly recognizing the contribution from *Q*_*non-mgt*_ nor the variation in economic optimum rate with prices.

### 2.4 Case study A: organic poultry and dairy farms in Brazil

This case study uses data from an organic dairy farm in Sao Paulo State, Southeast region of Brazil which uses poultry manure from a neighboring farmer to fertilize maize fields for production of feeds as green maize and maize silage. This is motivated by the fact that the manure and milking parlor effluent from the dairy enterprise are insufficient to fertilize the maize area and that due to the organic system, application of a mineral fertilizer is not allowed.

The dairy farm was monitored throughout the year 2016 with data available on a monthly basis (see supplementary information). Main annual data are provided in Table 1, while chemical characteristics of products and feeds are given in Table 2.

**Table 1:** Key data of the Brazilian dairy farm.

Number of lactating cows	average over year	67.4
Number of other cattle	average over year	47.3
Number of livestock units (LU, 500 kg)	average over year	95.4
Milk production	Mg milk per year	470
Manure excretion rate	kg manure per LU per day	55

**Table 2:** Chemical properties of product and feeds from the Brazilian dairy farm.

	Dry matter (DM)	Protein content	Nitrogen in protein	N Concentration	P Concentration
		g protein kg^−1^	g protein (g N)^−1^	g N kg^−1^	g P kg^−1^
Milk		37.4	6.38	5.86	0.9
Dairy manure				25	11.83
	**kg DM kg**^**−1**^	**g protein (kg DM)**^**−1**^	**g protein (g N)**^**−1**^	**kg N (kg DM)**^**−1**^	**kg P (kg DM)**^**−1**^
Poultry litter	0.8			0.029	
Grass		200	6.25	0.0320	
Maize grain		90	6.25	0.0144	
Soyabean meal		450	6.25	0.0720	
Maize silage	0.379	95	6.25	0.0152	0.0016

The dairy farm produced about 470 Mg milk, sold for a value of about US$ 224,000 from an average of 67.4 lactating cows (19 kg milk cow^−1^ day^−1^). The protein content of milk was 3.74%. We assumed an N content of 6.38 g N g^−1^ milk protein, and a P content of 0.9 g P kg^−1^ of milk (NEPA, 2012), giving a total of 2755 kg N in milk produced over the year. Manure excretion rate was 55 kg manure per livestock unit per day (LU, equivalent to 500 kg live weight; LW) totaling 1.9 kt manure year^−1^.

The feed mix for the lactating cows consisted of grass (20% protein), green maize (9% protein), soy meal (45% protein), and maize silage (9.5% protein). Feeding on rotational grass was possible during the wet season (September through April), and during the dry season (May through August) maize silage was fed. Over the year, a total of 12.6 t N was fed to the lactating cow herd (see Table 3).

**Table 3:** Feeding data for lactating cows (average n = 67.4).

		Wet season	Dry season	Farm total*
		Sep–Apr	May–Aug	
		kg DM day^−1^ cow^−1^	kg DM day^−1^ cow^−1^	kt N year^−1^
Feed				
Grass	10			5.0
Maize grain	8		4.5	2.4
Soyabean meal	1		4.5	3.9
Maize silage			10	1.3
**Total**				**12.6**

Most of the dairy manure was deposited on pasture land and the nutrients in the effluent from the milking parlor were insufficient for the cultivation of all the farm-grown feeds. Therefore, the farmer purchased poultry litter from a neighboring poultry farm, applying 20 t ha^−1^ on pasture and maize produced for silage. Thus, the farmer applied 464 kg N ha^−1^ year^−1^ on maize silage that compared to a harvest of only 230 kg N ha^−1^ year^−1^ (see Table 4 – results).

**Table 4:** On-farm feed cultivation.

	Area	Application poultry litter	Productivity	N content in harvested maize	P content in harvested maize
	ha	t ha^−1^	t ha^−1^	kg N ha^−1^ year^−1^	kg P ha^−1^ year^−1^
Rotational pasture	15	0			
Pasture	4	20			
Maize for silage	6	20	40	230	24

For the estimation of the allocation of emissions to the applied poultry litter we estimated a value for broilers of 0.65 US$ (kg poultry)^−1^ and a value of mineral fertilizer of 0.7 US$ (kg N)^−1^.

The poultry farmer had three barns with 3300 chickens in each barn. Two-thirds of the poultry litter produced was sold to the dairy farmer.

### 2.5 Case study B: laying operation

FAO (2016c, appendix 3) proposes a method for physical allocation of burden to eggs, meat and manure using the partitioning of the metabolizable energy (ME) into ME requirements for maintenance, growth, and production. This is used to calculate the Heat Increment of Feeding (HIF) to produce eggs, meat, and manure. The method is illustrated using an example of a laying operation with 1000 layers. Details on the calculation and background data for the example are found in FAO (2016c, appendix 3). The HIF-based allocation results in 46.5% for eggs, 27.4% for meat and 26.1% for manure, while the allocation between eggs and meat only (treating manure as a residual) is 63% for eggs and 37% for meat. The average spent hen weight was 3.3 kg; the mass of eggs produced in 100 weeks was 23.3 kg. We compare these results with an economic allocation and a mixed allocation approach. The economic allocation requires farm gate prices of cereals, mineral fertilizers, eggs, and poultry meat, which were obtained from the CAPRI database (for the year 2008) for EU-28. Other data required to obtain the value of manure versus the value of eggs and poultry meat are the N and P contents in each co-product, and the edible fraction of the poultry body mass, which are given in Table 5.

**Table 5:** A Summary for the calculation of the value of the co-products as illustrative examples for eggs, poultry meat and manure. See text above.

Item	Value	N	P	Unit	Note
**a) Eggs**					
Weight produced	23.3			kg	FAO (2016c), Appendix 3.
Nutrient content		0.018	0.002	kg (kg egg)^−1^	FAO, 2018a, FAO, 2018b, Appendix 6, Table 5, considering whole egg including shell
Nutrient in egg		0.43	0.04	kg	
Price	1182			Euro/t	CAPRI
**Value**	**27.5**	** **	** **	**Euro**	
**b) Poultry meat**					
Weight	3.3			kg	FAO (2016c), Appendix 3.
Carcass fraction	0.57				After Ramirez et al. (2012)
Nutrient content		0.028	0.004	kg (kg body mass)^−1^	FAO, 2018a, FAO, 2018b, Appendix 6, Table 6, average of reported values
Nutrient in body mass		0.092	0.013	kg	
Price	1379			Euro/t	
**Value**	**2.6**	** **	** **	**Euro**	
**c) Manure**					
Weight	12.8			kg FOM + UN	FAO (2016c), Appendix 3. Excretion of Faecal Organic Matter (FOM) and Urine Nitrogen (UN) at an intake of 84 kg of feed for a 100-weeks cycle of a layer with 3.3 kg cull weight and 23.3 kg of cumulative egg production.
Total nutrient intake		2.56	2.29	kg	
Total nutrient in manure		2.04	2.24	kg	
Nutrient content		0.159	0.174	kg (kg FOM + UN)^−1^	
Nutrient equivalent		44%	100%		Assuming loss of N in manure management systems (MMS) of 50% (based on values indicated in IPCC, 2006) and a higher volatilization rate upon application of 20% of manure versus 10% for mineral fertilizers. 100% nutrient equivalent assumed for P.
Fertilizer price		1037	409	Euro/t	
**Manure value**	** **	**0.9**	**0.9**	**Euro**

**Table 6:** Calculation of allocation factor for poultry emissions from maize silage via applied poultry litter.

		Poultry	Poultry litter	Mineral fertilizer	
Application of poultry-N	t DM ha^−1^ year^−1^		16		
Losses as NH_3_	%		20	**20**	d
Losses in leaching	%		13	**13**	e
N available for crops			323	230	
N uptake by maize	kg N ha^−1^ year^−1^		230	**230**	
Nutrient equivalent		**100%**	30%	30%	
Application of poultry-N	kg N ha^−1^ year^−1^		**464**		
Equivalent application of mineral fertilizer	kg N ha^−1^ year^−1^			**330**	
Manure applied in excess of need	kg N ha^−1^ year^−1^		134	** **	
Manure production from broiler chickens	kg N (1000 kg poultry)^−1^ day^−1^	**1.1**	** **	** **	a
Price	US^e^ (kg poultry, kg N in poultry litter or mineral fertilizer)^−1^	**0.65**	**1.5**	**0.7**	b
Value	US^e^ ha^−1^	**1127**	**710**	**241**	c
**Value of poultry litter according to nutrient equivalents**	**%**	** **		**18%**	
**Value of poultry litter according to price paid by the dairy farmer**	**%**	** **	**39%**	** **	

Notes.

a Source: IPCC 2006, Table 10.19.

b Price of poultry estimated. The price paid for the poultry litter was R$ 115 per ton of wet poultry litter. Average price of urea in Brazil in2016 was R$ 1,100,00 per ton or US$ 355 per ton = 1,26 US$ (kg N)^−1^ (exchange rage 0.31 US$ per R$).

c Revenue from broiler chickens based on a price for chicken meat in Brazil of 0.65 US$ (kg poultry)^−1^ and a share of poultry litter sold of 67%.

d Volatilization rates for manure as per default IPCC (2006); volatilization rate for mineral fertilizer assuming application of urea (45% N). Emission factor from EEA (2016), Chapter 3D-Table 3.2 EF for NH_3_ emissions from fertilizers (in g NH_3_ (kg N applied)^−1^) for urea in warm climate.

e Leaching of manure considering the climatic, soil and agricultural practices in the farm. We assumed the same leaching rate for mineral fertilizer.

This example has no ‘crop farmer’ who buys the manure but is illustrating that nutrient equivalents could be obtained in principle also without knowing specifically where the manure is applied.

## 3 Results

### 3.1 Case study A: organic poultry and dairy farms in Brazil

Table 6 shows the data required to calculate the allocation factor to be applied to sold poultry manure for allocating emissions from poultry production (in housing and manure management before selling) to the dairy farm. The calculation assumes losses of ammonia (NH_3_) of 20% for both poultry litter (IPCC, 2006) and urea (EEA, 2016) and losses via leaching of 13% (estimated considering the climatic, soil and agricultural practices in the farm for poultry litter and assuming the same leaching rate for mineral fertilizer). With a crop uptake of 230 kg N ha^−1^ year^−1^, this could have been met by application of 330 kg N ha^−1^ year^−1^ of mineral nutrient equivalents (using the pragmatic approach). Applying a price of 0.7 US$ kg^−1^ of N in mineral fertilizer, the value of the nutrient equivalent is 241 US$.

It is known that the poultry farmer sells two thirds of the poultry litter to the dairy farmer. Based on manure production of 1.1 kg N (1000 kg of broiler chickens)^−1^day^−1^ (IPCC, 2006) to calculate the amount of poultry per hectare-equivalent and a price of 0.65 US$ kg^−1^ of chicken sold, the revenue for chicken meat is US$ 1127.

The poultry farmer has thus two products from the poultry: poultry meat with a revenue of US$ 1127 for each ha of maize silage production the manure was applied to, and manure for an equivalent mineral fertilizer value of US$ 241 ha^−1^ of maize silage production where the poultry litter was applied. This results in an economic allocation factor of 18%. However, the real price that was paid for the poultry litter was US$ 710 ha^−1^. The dairy farmer thus not only bought about 40% more poultry litter than would be required to achieve the same yield, but paid also a price that was almost three times as high as the price the farmer would have had to pay for mineral fertilizers.

### 3.2 Case study B: laying operation

Using the data from Table 5, the economic calculations result in allocation of 6% of emissions to manure, 94% to eggs (86%) and meat (8%) (Table 7). The allocation takes into consideration all value that manure gives to the farmer for crop production, which in this example is the sum of the economic values of N and P. However, other values could be considered as well (carbon, soil structural benefit), as long as the benefit can be monetized. The allocation amongst eggs and meat varies depending on whether the physical allocation factors developed by FAO (2016c) are used, or all allocation factors calculated based on economic allocation. Table 7 compares the result of both methods with a ‘mixed’ approach (see footnote of Table 7) and an economic approach considering manure as residual.

**Table 7:** Allocation factors of the poultry system in the example over eggs, poultry meat and manure on the basis of economic allocation between manure and food products and physical allocation based on heat increment for feeding (FAO, 2016c).

	Value (Euros; Table 5)	Allocation based on the heat increment for feeding	Mixed allocation method^a^	Economic allocation based on fertilizer value	Economic allocation considering manure as residual
Eggs	27.5	0.465	0.59	0.86	0.91
Poultry meat	2.6	0.274	0.35	0.08	0.09
Manure	1.8	0.261	0.06	0.06	0

a For the mixed allocation method, economic allocation is used for manure versus other co-products, and bio-physical allocation for eggs versus poultry meat.

Thus where manure is considered as co-product, 6% of burden is allocated to the crop it is applied to (when it is applied to land). To determine if the application of manure is to be considered as waste, additional information is required, such as the sources of other inputs to the land including atmospheric deposition, biological fixation, and mineralization of soil organic matter or use of inputs from previous years (e.g. crop residues) (but not the input of mineral fertilizers), and the maximum amount of nutrients that should be applied at the economic and physical optima.

## 4 Discussion

Recognizing the nutrient value in manure and thus treating manure as a co-product may encourage the livestock farmers to ensure that nutrients in manure at excretion are not lost during manure management and storage as this will directly decrease the nutrient equivalent value of his/her product. Additionally, it is in the interest of livestock farmers to ensure appropriate use of the nutrients, i.e. no application rate in excess of crop needs. For farmers living in regions of high livestock density as is the case for many Brazilian farmers, there is generally an oversupply of manure, which leads to manure being wasted, unless it is processed and/or transported to regions with demand for manure.

However, economic incentives for efficient manure management practices and efficient nutrient use depend on the existence of a price for the external effects allocated, so we might hypothesize what would happen if GHG emissions from agriculture would be included in a carbon trading scheme? In practice, livestock farmers would need to be certified for the sustainable use of the manure in order to be able to get credits for the GHG emissions. This context holds also for the instances where manure is used for various other purposes including biogas generation (Amjid et al., 2011), biomass fuel (Roy et al., 2010), feeding of animals and fish (Negesse et al., 2007) and production of insects for feeds or foods (Hussein et al., 2017). In these situations, an approach similar to the one explained here could be developed, based on the value as a fuel or feed.

While cropping farmers are interested in the nutrient content of manures, there are also GHG emissions associated with their use. A farmer may only purchase manure if the GHG burden is equal or lower than the equivalent emissions from mineral fertilizer, or the price is lower so that the additional cost for GHG emissions is compensated. This view applies to regions where livestock density and feeding practices lead to oversupply of nutrient. In many low income countries such as those in Sub-Saharan Africa, manure is often the only source of nutrient for crop production and farmers often keep livestock of this reason (Rufino et al., 2007, 2006).

If manure carries a higher GHG burden it will be less attractive to cropping farmers and would otherwise remain in the livestock supply chain, or need to be disposed of. The situation potentially changes if carbon sequestration associated with manure use is taken into consideration. In this case, it might be attractive for the crop farmer given the evidence that continuous applications of manure can increase soil C and N stocks (Zavattaro et al., 2017).

The value of manure, when applied at agronomic rates, may extend beyond nutrient replacement value to include soil quality improvements. These benefits are difficult to quantify economically but include natural resource and productivity value. Application of manure can influence the soil biological, physical and chemical environment with impacts on crop productivity, including benefits of macro-aggregate formation with reduced soil loss and runoff over several seasons (Graham et al., 2014; Wortmann, 2013; Wortmann and Shapiro, 2008). An extensive literature review of 141 studies comparing manure substitution for fertilizer revealed that manure had average reductions of 26% and 29% in nitrogen loss to surface and ground water, respectively (Xia et al., 2017).

What is the correct price for manure? In case study A, the farmer paid more per kg N in poultry manure than one would have to pay for mineral fertilizer. However, since organic farming prohibits the application of mineral fertilizer, manure is a scarce nutrient resource for such a high-productive crop and the farmer was thus prepared to pay a high price. In that case, it seems appropriate to allocate the higher share of 39% of poultry emission to maize silage production, which will increase the footprint of the milk as the main product at the dairy farm. On that farm, the application of manure was in excess of crop needs. We estimated that for a crop uptake of 230 kg N/ha, a quantity of more than 450 kg N/ha in poultry manure was purchased and applied. A large part of the applied manure was ‘wasted’. In Section 2.1 we argued that wasted manure (i.e. applied in excess of crop needs or not used at all for any benefit) is to be considered as ‘waste’ thus all emissions remain (or are ‘given back’) to the main product(s) of the farm where the manure was produced, i.e. to poultry meat. This might seem ‘counter-intuitive’ as the dairy farmer paid good money for the ‘wasted’ manure, but the associated emissions go back to the poultry farmer. This leads to the strange situation in this case study that 18% of emissions from poultry and poultry manure management before selling of the manure is allocated to milk, but 29% (i.e. 134 kg N ha^−1^excess of the total 464 kg N ha^−1^ applied) of emission caused by management of the manure on the dairy farm and from application of the manure to the maize fields is allocated ‘back’ to poultry meat. Unfortunately, there is no information on the management and emissions of the poultry farm.

An alternative method for estimating the allocation of poultry emission to the dairy milk is to use the price that has been paid. This would be the ‘normal’ approach for any economic allocation procedure, but prices may be distorted and this would not give reasonable results in most cases. The pressure of getting rid of the manure due to environmental restriction for its application can lead to the fact that a price is paid that is more driven by the opportunity cost of having to dispose of the manure in other sustainable ways and could thus even be negative in case additional costs for the transport of the manure to the selling location occur to the farmer. In many cases though, manure is sold below the price of its fertilizer value and the price paid for manure can therefore not be used generally for the allocation of emissions.

The approach developed here is motivated to incentivize farmers producing manure to proper use of the nutrients beyond their own farm if they want to minimize the footprint of their products. If responsibility is transferred to the crop farmer, the livestock farmer has less motivation to ensure proper use of the manure. In many cases, the price paid for the manure will be lower than the nutrient equivalent value, and in some cases there might not even be a crop product to which the waste could be allocated to. One way to incentivize change may be to assign the emissions of the wasted manure back to the livestock system. The crop farmer has less interest in using the manure carefully, although may be affected by other factors (e.g. effects on water quality or cost of fertilizer if manure nutrient value is ignored), but needs to ‘certify’ proper use to the livestock farmer. Thus, this approach engages both partners and makes both liable if the manure is applied excessively.

Empirical evidence from crop-livestock mixed farming in Kenya (Castellanos-Navarrete et al., 2015) shows that manure is often the only source of nutrients for crops, representing a modest amount of the total crop uptake (16 kg N per ha), and about 300 kg C per ha to the soil. Yet, this addition of nutrients is crucial to sustain the production of food crops in the absence of other sources of nutrients (Castellanos-Navarrete et al., 2015). A recent study by Rurangwa et al. (2017) suggested that, in Rwanda, cattle manure is sold to crop farmers that grow vegetables, but its price is variable by district. For example, in the Kamonyi district, a pit of 5 t of manure, which contained 45 kg N, 25 kg P and 65 kg K cost 27 US$, whereas in Bugesera, it cost 13.45 US$, despite containing more nutrients, i.e. 90 kg N, 10 kg P and 70 kg K. Considering N only, the difference in manure price was 4-fold between these districts. The overall price, however, was lower than the urea market price in Rwanda, estimated at 1.41 US$ kg N^−1^.This indicates that in some countries, manure is treated as a valuable resource, but uncertainty in the benefit means that its price is variable and consequently cheaper than fertilizer.

In the Brazilian case study, we accounted only for the value of nitrogen in the poultry litter, while case study 2 accounted for N and P in manure. However, co-application of N and P in manure might lead to a situation of a high nutrient equivalent value for one nutrient and no fertilizer value for another nutrient. In several countries manure was seen as a source of N mainly but had led to excessive accumulation of P in soils resulting in eutrophication of aquatic and terrestrial ecosystems. In this case, application of manure should be based on the required rate of the most limiting nutrient (P) in order to avoid the negative consequences related to over-application of that nutrient. The approach proposed in this paper needs therefore to be seen as one of several measures that needs to be combined for example with application limits to avoid food safety risks related to pathogens, heavy metals or other substances, and ensure environmental stewardship. Individual nutrients might be given value only if the soil test concentration is at levels consistent with recommendations for crop response from nutrient supplementation, taking into consideration multiple cropping years for less mobile nutrient such as P. The time window for evaluating manure nutrient value vs. waste should be specific for all nutrients commonly supplemented in a fertility program (most commonly N and P, possibly K, S, and Fe).

## 5 Conclusion

We developed a relatively simple methodology that calculates the allocation of emissions from livestock production systems to manure and other animal products such as eggs, milk, or meat. As manure is often traded at a price that is not necessarily linked to its fertilizer value, the approach quantifies the value of an equivalent application of mineral fertilizers, which the farmer would apply in order to achieve the same crop yields. We believe that embedding a part of the emissions from livestock supply chains in the manure that is used as a fertilizer on crops outside the supply chains might contribute to increased awareness of the environmental effects of emissions associated with manure and consequently more sustainable management of manure. However, this can work only if the cost of the externalities caused by manure (contribution to global warming, health impacts through air pollution, coastal eutrophication, to name a few) are priced into their causes, the emissions of GHGs and losses of nutrients to atmosphere and waters. Overall, the approach developed here could potentially contribute to more awareness of the consequences of excess manure production and ultimately to improved management. Improved manure management could increase overall yield which would be important in countries/regions where nutrients are a limiting valuable resource, as noted in some African countries. Greater use of manure with less waste could lead to increased circularity and reduced fertilizer requirement.

## Contributions

AL conceived the idea of the paper; AL, SL and AU contributed equally to the writing of the manuscript; JP contributed with data and calculations for the Brazilian case study; all co-authors contributed with discussions of the approach and to the writing of the manuscript.

## Figures and Tables

**Table:** 

${\text{α}}_{\text{s}}$	dimensionless	Allocation factor for burden to a co-product *s*
$C_{min,nut}$	US$ (kg Nutrient)^−1^	Cost of nutrient *nut* supplied with nutrient source type *min* (min = mineral fertilizer)
$C_{man,nut}$	US$ (kg Nutrient)^−1^	Cost of nutrient *nut* supplied with the manure applied and considered as co-product for nutrient *nut*
$E_{s}$	X yr^−1^	Burden from source *s*. The kind of emissions considered depends on the nutrient and the context of the study. Here we assume that the approach is used to allocate total greenhouse gas emissions, and X is therefore kg CO_2_eq, but the approach is applicable also to other types of burden.
${\text{f}}_{\text{eq}}$	dimensionless	Nutrient equivalent, ratio of mineral fertilizer application at the economic optimum and manure application rate yielding the same N uptake.
$f_{discount}$	dimensionless	Discount factor for accounting for reduced benefit of manure application for rates between the economic optimum and the physical maximum for calculating the total value of the applied manure.
$Frac_{loss,t,nut}$	dimensionless	Fraction of nutrient *nut* of type *t* lost to the atmosphere (loss = *atm*) or to the hydrosphere (loss = *hyd*). Losses are considered to be sequentially, with losses to the atmosphere occurring before losses to the hydrosphere. $Frac_{loss,hyd}$ QUOTE needs therefore to be applied to the nutrients that are remaining after accounting for losses to the atmosphere.
$Q_{\min\left[,nut\right]}$	kg Nutrient ha^−1^ yr^−1^	Application rate of the nutrient *nut* with fertilizer type *min* (min = mineral fertilizer)
$Q_{man\left[,nut\right]}$	kg Nutrient ha^−1^ yr^−1^	Application rate of the nutrient *nut* with nutrient source type *man* (min = manure)at the point *p.*
$Q_{t\left[,nut\right]\left[,p\right]}$	kg Nutrient ha^−1^ yr^−1^	Flux rate nutrient *nut* of type *t* from at the point *p.*
$Q_{full,nut}$	kg Nutrient ha^−1^ yr^−1^	The amount of manure valued as a co-product with the full fertilizer price for nutrient *nut*
$Q_{low,nut}$	kg Nutrient ha^−1^ yr^−1^	The amount of manure valued as a co-product with lower than fertilizer price for nutrient *nut*
$Q_{coprd,nut}$	kg Nutrient ha^−1^ yr^−1^	The amount of manure valued as a co-product considering $N_{full,nut}+\frac{1}{2}N_{half,nut}$ QUOTE for nutrient *nut*
$NUE_{min\left[,nut\right]\left[,p\right]}$	dimensionless	Share of nutrient *nut* supplied with nutrient source type *min* (min = mineral fertilizer) at the point *p* that is taken up by the plant as a whole, including crop residues, plus possible accumulation of nutrient in the soil.

## Glossary

Economic optimum: The economic optimum is the nutrient application rate $N_{ecopt}$ at which the marginal cost of additional nutrient application (e.g. the cost of the next kg of applied fertilizer) equals the marginal revenue induced by the additional nutrient application (that is the revenue obtained from increased harvest induced by the increased nutrient supply). Nutrient application rates $N>N_{ecopt}$ lead to a negative marginal cost for the farmer
Non-manageable nutrient sources: This includes nutrients supplied by atmospheric deposition, biological nitrogen fixation or mineralization of soil organic matter. These nutrients depend (partly) on farm management practices, but cannot be precisely steered by the farmers and come at no (additional) cost
Nutrient equivalent: The nutrient equivalent $f_{eq}$ is the ratio of mineral fertilizer application at the economic optimum and manure application rate yielding the same N uptake
Physical optimum: The physical optimum is the nutrient application rate $N_{mx}$ at which additional supply of nutrients do not lead to an additional plant nutrient uptake. Practically, the physical optimum is defined as the nutrient application rate at which the plant nutrient uptake reaches a certain threshold (e.g. 95% or 99% of the maximum plant nutrient uptake)

## Symbols used

**IndicesExplanations see glossary and/or symbols used.**
*i*: Fields over which manure is applied
*p*: Point of application rate – optional index for supply rates of nutrientsp∈{ecopt,mx,standard}
*t*: Type of nutrient flow t∈{input,output,plant,non−mgb,ssc,bnf,atmdep,min,man,coprd,waste}
*s*: Source of burden/emissions. s∈{man,lvst,livstock_total,crop}
atmdep: Atmospheric deposition
*bnf*: Biological nitrogen fixation
*coprd*: Manure considered as a co-product
*Crop*: Crop (system)
*ecopt*: Economic optimum
*full*: Manure valued with full fertilizer price
*low*: Manure valued with lower than fertilizer price
*input*: Input of a nutrient
lvstk: Livestock (system)
lvstk_total: Livestock system including a share of burden from other systems allocated to the livestock system
*mx*: Physical optimum
*min*: Mineral fertilizer
*man*: Manure
*non-mgb*: Non-manageable sources of nutrients
*nut*: Nutrients. In this study nut∈{N,P} , but other nutrients or beneficial properties of manure could be included as well
*plant*: Uptake of nutrients into the plant biomass
*output*: All useful outputs of nutrient
*ssc*: Soil stock changes. Accumulation of nutrients in the soil are considered as (useful) output, while depletion of nutrients in the soils are negative outputs.
*standard*: Default of representative loss rates of nutrients, obtained from official guidelines or measurement/modelling at similar conditions
*surplus*: Soil nutrient surplus
*waste*: Manure considered as waste
