# Supplementary material for: The value of manure - Manure as co-product in life cycle assessment
Source: J Environ Manage. 2019 Jul 1;241:293–304. doi: 10.1016/j.jenvman.2019.03.059 (PMC6531380; doi:10.1016/j.jenvman.2019.03.059)
Supplement: Multimedia component 1 [file mmc1.docx]

The value of manure - manure as co-product in life cycle assessment

Supplementary Information: Monthly data collected at the Dairy Farm in 2016.

|  |  | **Jan** | **Feb** | **Mar** | **Apr** | **May** | **Jun** | **Jul** | **Aug** | **Sep** | **Oct** | **Nov** | **Dec** | **TOTAL** |
| --- | --- | --- | --- | --- | --- | --- | --- | --- | --- | --- | --- | --- | --- | --- |
| Poultry Litter | R$ | 30000 | 11500 | 1700 | 9360 |  | 37840 | 14400 |  | 2000 |  |  |  | **106800** |
| Poultry Litter | Ton | 250 | 96 | 14 | 78 |  | 344 | 131 |  | 19 |  |  |  | **932** |
| Poultry Litter (dry matter) | Ton | 200 | 77 | 11 | 62 |  | 275 | 105 |  | 15 |  |  |  | **746** |
| Poultry Litter - N | kg | 5800 | 2227 | 325 | 1810 |  | 7981 | 3039 |  | 441 |  |  |  | **21622** |
| Poultry Litter - P | kg | 2444 | 938 | 137 | 763 |  | 3363 | 1281 |  | 186 |  |  |  | **9111** |
| Lactating Cows/day | nº | 70 | 63 | 69 | 72 | 77 | 81 | 68 | 62 | 63 | 56 | 55 | 73 | **67** |
| heifers, calves, dry cows | nº | 54 | 53 | 54 | 55 | 36 | 49 | 52 | 40 | 35 | 43 | 45 | 51 | **47** |
| Animal Unit (500 kg) | AU | 100 | 94 | 101 | 108 | 95 | 107 | 96 | 83 | 88 | 89 | 90 | 93 | **95** |
| Manure excretion | Ton | 166 | 155 | 167 | 178 | 157 | 177 | 158 | 137 | 145 | 146 | 148 | 153 | **1889** |
| Manure N content | Ton | 4 | 4 | 4 | 4 | 4 | 4 | 4 | 3 | 4 | 4 | 4 | 4 | **47** |
| Manure P content | Ton | 2 | 2 | 2 | 2 | 2 | 2 | 2 | 2 | 2 | 2 | 2 | 2 | **22** |
| Effluent/month | L | 112840 | 91728 | 111228 | 112320 | 124124 | 126360 | 109616 | 99944 | 98280 | 90272 | 85800 | 117676 | **1262040** |
| N in the effluent | kg | 91 | 74 | 90 | 91 | 100 | 102 | 89 | 81 | 80 | 73 | 69 | 95 | **1021** |
| P in the effluent | kg | 5 | 4 | 5 | 5 | 5 | 5 | 5 | 4 | 4 | 4 | 4 | 5 | **53** |
| Milk Production | R$ | 60722 | 52408 | 55789 | 61653 | 68354 | 70762 | 67864 | 63019 | 58754 | 55058 | 49797 | 58930 | **723107** |
| Milk Production | kg | 39639 | 34427 | 36556 | 40213 | 44409 | 45906 | 41790 | 41075 | 38401 | 36099 | 32803 | 38519 | **469837** |
| Milk Crude Protein | % | 4 | 4 | 4 | 4 | 4 | 4 | 4 | 4 | 4 | 4 | 4 | 4 | **4** |
| Milk N | kg N | 231 | 199 | 209 | 226 | 263 | 271 | 250 | 245 | 225 | 216 | 193 | 226 | **2753** |
| Milk P | Kg P | 36 | 31 | 33 | 36 | 40 | 41 | 38 | 37 | 35 | 32 | 30 | 35 | **423** |
| Milk per cow | kg | 18 | 20 | 17 | 19 | 19 | 19 | 20 | 21 | 20 | 21 | 20 | 17 | **19** |
